# A Bayesian model of legal syllogistic reasoning

**DOI:** 10.1007/s10506-023-09357-8

**Published:** 2023-04-24

**Authors:** Axel Constant

**Affiliations:** 1 School of Engineering and Informatics, The University of Sussex, Chichester I, CI-128, Falmer, Brighton, BN1 9RH UK; 2 Centre de Recherche en Éthique (CRÉ), University of Montreal, Montreal, Canada

**Keywords:** Computational law, Legal cognition, Bayesian legal reasoning, Legal syllogism, Bayesian network, Entropy

## Abstract

Bayesian approaches to legal reasoning propose causal models of the relation between evidence, the credibility of evidence, and ultimate hypotheses, or verdicts. They assume that legal reasoning is the process whereby one infers the posterior probability of a verdict based on observed evidence, or facts. In practice, legal reasoning does not operate quite that way. Legal reasoning is also an attempt at inferring applicable rules derived from legal precedents or statutes based on the facts at hand. To make such an inference, legal reasoning follows syllogistic logic and first order transitivity. This paper proposes a Bayesian model of legal syllogistic reasoning that complements existing Bayesian models of legal reasoning using a Bayesian network whose variables correspond to legal precedents, statutes, and facts. We suggest that legal reasoning should be modelled as a process of finding the posterior probability of precedents and statutes based on available facts.

## Introduction

Bayesian approaches to legal reasoning often rely on legal heuristics to capture the way in which one reasons about the causal relations between evidence and verdicts, or “ultimate hypotheses”(Neil et al. 2019) in legal proceedings. Bayesian approaches to legal proceeding are concerned with providing a normative assessment of the way one ought to reason about the strength of legal evidence. Such approaches are relevant, for instance, during the preparation of an expert report when dealing with conflicting hypotheses (e.g., the probability that oil or gasoline caused the fire). Expert reports, which in some jurisdictions are treated as testimonies whose strength will be evaluated by the judge, are then used in support of the allegations. The writing of allegations requires the articulation of substantial laws (e.g., the rules establishing the burden of proof in tort law) and of the facts supported by the evidence. Accordingly, legal operations can be split in two general categories: procedural law operations (i.e., the administration of evidence and the application of modes of proof to identify the facts relevant to the allegations and defend those allegations in front of the court), and substantial law operations (i.e., the analysis of rules as applied to the facts at hand to give a substance to the allegations). This distinction is evident in many common law jurisdictions where practitioners will tend to specialize in either “litigation law” (i.e., dealing with the procedural aspect in front of the court), or legal counsel (i.e., dealing with the substantial aspect upstream of litigious situations). Accordingly, one ought to make a distinction between Bayesian approaches to legal proceeding and Bayesian approaches to overall legal reasoning. Bayesian approaches to legal proceedings solely seek to model the procedural steps involved in the preparation of a judicial decision (e.g., weighing of the evidence by an expert witness). In turn, an overall approach to Bayesian legal reasoning should capture the broad scope of the reasoning that underwrites the way practitioners and the legal profession cognize about the law. Because standard Bayesian approaches to legal reasoning solely focus on the component of the legal procedural operations concerned with the preparation of the evidence needed to prove the facts that support allegations, they miss an important part of the overall process of legal reasoning, which involves many other processes mediating the process of moving from evidence to the verdict (e.g., those of substantial law analysis).

This paper proposes a Bayesian approach to substantial legal reasoning that differs from standard approaches, by focusing on the way facts, once proven, are articulated with rules to yield a decision. Despite having a different scope, our approach can be viewed as extending the project of standard Bayesian approaches by providing a model that covers substantial legal reasoning. As in the case of standard approaches, the Bayesian model employed here is well suited to deal with uncertainty in legal reasoning (Fenton et al., 2012). As we will see, in substantial reasoning, uncertainty arises from the interpretation of case law, and from statutes that may lack the precision needed to fit the specific facts at hand. We frame our approach to Bayesian legal reasoning as Bayesian “legal cognition”. The term cognition refers to the processes of knowing, reasoning, judging, or solving problems. We use the term “cognition” instead of “reasoning” for two reasons. First, it allows us to stress the fact that the approach outlined in this paper could be used to model both the way a *legal professional* – or an artificial version of such a professional – cognizes about the law, as well as the way the *legal profession* reasons about the law in common law and civil law systems. Legal cognition as presented here corresponds to the inference process from relevant and demonstrated facts (i.e., evidenced facts) to applicable statutory or jurisprudential rules, the assemblage of which constitutes the justification for a final decision. For instance, penal and civil liabilities are cumulative concepts that obtain when a subset of legal criteria established by the section of a code, a statute, or by a judicial decision is met (e.g., *mens rea* and *actus reus* of an infraction from the Canadian criminal code, or the fault, cause and injury in torts law, Sect. 1457 of the Quebec Civil Code). In legal reasoning, there is no direct causal link between the ultimate hypothesis, or final decision, and the evidence, such as assumed by typical Bayesian models of legal proceedings. The final decision just is an emergent property of the assemblage of applicable and evidenced rules, which is achieved through substantial reasoning, or here legal cognition. Second, and perhaps more importantly, the use of the term “legal cognition” is a practical way to distinguish our approach from the standard ones. However, note that given the scope of this paper, we could have chosen to distinguish our approach by referring to it as a Bayesian approach to “substantial” reasoning in contrast to “procedural” reasoning.

We illustrate the Bayesian approach to legal cognition presented in this paper using a toy model that replicates types of reasoning patterns that lawyers, judge, and students alike use to solve litigious situations. These patterns can be found in any judicial decision, and primitive forms of those are at the basis of undergraduate and professional legal education. These patterns refer to the legal syllogism, which is a standard of legal positivism (Hart 2012; Kelsen 1967). In its most basic form, the legal syllogism involves stating a rule (e.g., All B are C) and qualifying the facts at hand (e.g., A is B) to arrive at a conclusion (e.g., therefore A is C). The toy model of legal cognition that we present in this paper implements legal syllogistic reasoning as a process of inference in a simple Bayesian network whose unknown random and known variables represent various elements articulated in legal syllogistic reasoning; the way precedents are used to interpret statutory law, and how statutory law is used to qualify the facts at hand to yield a conclusion. Section 1 of this paper discusses syllogistic reasoning in legal practice. Section 2 formalizes syllogistic legal reasoning with a decision tree, which in Sect. 3, will be reinterpreted as a Bayesian network. We conclude with a note on the relevance of the proposed approach in the field of artificial intelligence and the law.

## The legal syllogism

The legal syllogism is a liberal form of categorical syllogism whereby an implication relation between a minor premise and a major premise is used to infer a conclusion. The legal syllogism leverages the transitivity property of first-order logic (e.g., “If A = B, and if B = C, then A = C”) to structure and diagnose legal arguments. A canonical example of categorical syllogism using transitivity would be as follows: Socrates is a man (minor, or A is B); all men are mortal (major, or all B are C); therefore, Socrates is mortal (conclusion, or A is C); or Barnewall called Adolphus a liar (minor); any person who calls another person a liar has a duty to pay $50 to that other person (major); thus, Barnewall has a duty to pay $50 to Adolphus (conclusion)(Gardner 2007).

We say that the legal syllogism is “liberal” in the sense that it is not meant to be held to the same standards as those that a logician would expect from syllogistic reasoning(cf. d’Almeida 2019). The legal syllogism just is a heuristic guide to the application and analysis of legal sources. In practice, the legal syllogism is not rigidly applied, as sophisticated logical errors are not likely to be invoked to overturn a decision. Legal practitioners are not logicians. Rather, the legal syllogism will serve as a rough guide to analyse legal arguments, and to diagnose whether the law was correctly applied in the parties’ arguments. Judges routinely appeal to the notion of a syllogism in order to assess the claimants and defendants’ arguments in that way. For instance, in a decision of Canada’s Quebec Court of Appeal – *Pharmascience inc. v. Option Consommateurs*, the Court dismissed the appeal of the appellant (Pharmascience inc.) on the basis that her argument confused the purpose of the rules invoked in the argument. To arrive at that conclusion, the Court reconstructs the syllogism in the appellant’s argument as follows (see the breakdown in Table 1):

“*The appellant’s position is based on the following syllogism. Instituting a class action requires the applicant to obtain judicial authorization, which requires a judicial decision whereby a judge recognizes that the motion meets the mandatory conditions set out in article 1003 C.c.p.. Furthermore, the fundamental right to a hearing before an independent and impartial tribunal, set out in section 23 of the Charter of human rights and freedoms (hereinafter the Charter), includes an obligation on the part of all applicants to establish the facts underlying the exercise of a private or civil recourse against a third party and, inversely, the fundamental right to demand evidence supporting such facts from the third party before a defence is produced. These are the two major premises presented by Pharmascience. The minor premise can be stated as follows: the amendment to article 1002 C.c.p. absolved the applicant from the obligation to prove the facts supporting its legal claims by removing the requirement of supporting the motion with an affidavit. The appellant therefore concludes that the amendment to article 1002 C.c.p. violates article 23 of the Charter. It adds that the proviso authorizing the court to admit relevant evidence cannot save the provision. This reasoning leads Pharmascience to arrive at the following conclusions:*

**Table 1:** Appellant’s argument in Pharmascience inc. v. Option Consommateurs

Minor	**-E (“- “ means that E is “false”)**: The amendment to 1002 C.c.p. for certification absolved from proving the facts justifying her claims	“The amendment to article 1002 C.c.p.. absolved the applicant from the obligation to prove the facts supporting its legal claims by removing the requirement of supporting the motion with an affidavit”
Majors	**If A then B, and if B then C**: A class action (A) requires certification (B), and certification (B) requires a judicial decision over the certification criteria of 1003 C.c.p. (now 575 C.c.p.) (C)	“Instituting a class action requires the applicant to obtain judicial authorization, which requires a judicial decision whereby a judge recognizes that the motion meets the mandatory conditions set out in article 1003 C.c.p..”
	**If C then D, and if D then E**: The right to independent and impartial hearing (D) requires that the applicant provides the facts justifying her claim (E)	“Furthermore, the fundamental right to a hearing before an independent and impartial tribunal, set out in Sect. 23 of the Charter of human rights and freedoms (hereinafter the Charter), includes an obligation on the part of all applicants to establish the facts underlying the exercise of a private or civil recourse against a third party and, inversely, the fundamental right to demand evidence supporting such facts from the third party before a defence is produced.”
Conclusion	**If -E, then -D,-C,-B,-A**: The class action must be certified, which involves a judicial decision, which itself supposes that facts were presented to the court, so as to fulfil the claimant’s obligation to present relevant facts. Since 1002 C.c.p. absolves from presenting relevant facts, 1002 C.c.p. violates Sect. 23 of the Charter and is unconstitutional	“The appellant therefore concludes that the amendment to article 1002 C.c.p. violates article 23 of the Charter.”

- *article 1002 C.c.p. must be declared invalid. This would in turn render article 1003 inapplicable and the Quebec class action regime inoperable;*
- *the action of the applicants, respondents on appeal, must be dismissed*.”(“Pharmascience Inc. v. Option Consommateurs,” 2005 par. 16.)

By fleshing out the appellant’s argument, the Court manages to identify where the argument fails; though, not based on faulty logical reasoning, but rather based on the reading of the law proposed by the appellant:

“*In my view, this reasoning is erroneous. For one, it confuses the nature and purpose of the motion for authorization of the institution of the class action and the consequent ruling with the action proper and the judgment on its merits. In addition, it misapprehends the scope of both section 23 of the Charter and the amendment to article 1002 C.c.p..*”(“Pharmascience Inc. v. Option Consommateurs,” 2005 par. 17)

In legal practice, the goal of syllogistic reasoning is to build arguments, the structure of which will allow the Court to reason backward, from consequences (i.e., facts) to causes (i.e., rules) to diagnose where the application of rules to the facts might have been misunderstood. This is what the Court does in the above quotes as it reconstructs the syllogism underlying the appellant’s argument and shows where the law was misinterpreted. In short, the syllogistic form of the argument is there to allow one to navigate the argument, not to give the argument a substance. The substance comes from the correct application of rules, not from the logical articulation per se, beyond mere transitivity. We now turn to a presentation of the formal structure of syllogistic reasoning, using a tree like structure amenable to Bayesian network modelling.

## The formal structure of the legal syllogism

In a legal syllogism, the rules are used to qualify facts that are relevant to the matter at hand. The relevant facts, in turn, are those that conform to the rules of evidence (e.g., hearsay makes a fact not relevant). The facts function as consequences of the law, or the rules, in the sense that they are events that emanate from the law. Pure facts alone have no meaning under the law, and are always conditioned on a rule (e.g., a “person” just is a human with civil rights (Civil Code of Quebec 1991 ss. 1)). The rules of evidence, in turn, allow for the transformation of mere facts into facts that can be qualified under the law (e.g., the birth certificate proves that that person was born from these parents on that day (Civil Code of Quebec 1991, ss. 2813)). Accordingly, to understand the formal structure of the legal syllogism, one ought to clearly distinguish between the way the law deals with rules and the way the law deals with evidenced facts. This is an important distinction between the Bayesian models of legal reasoning, which often assume that legal reasoning operates from evidence to the final verdict (Fenton et al. 2016), and the Bayesian model of legal cognition discussed here, which is about the qualification of evidenced facts.

The analysis of facts as per the rules of evidence and the qualification of facts under substantial rules are two distinct processes. The former allows for the processing of relevant facts, and the latter allows for the reaching of a verdict based on applicable rules. The separation of substantial and procedural processes in judicial proceedings is evidenced by the fact that syllogistic reasoning as applied to rules and facts can operate independently, for instance, as in the case of class action certification. For instance, in Canadian law, at the stage of the certification, the judge must “refrain from weighing the evidence and thereby intruding into the sphere of the trial judge”(“L’Oratoire Saint-Joseph Du Mont-Royal v. J.J,” 2019, par. 210), and the facts are “assumed to be true as long as they are sufficiently specific and concrete to make it possible for the defendants to know the allegations against them and for the court to assess the quality of the legal syllogism”(“L’Oratoire Saint-Joseph Du Mont-Royal v. J.J,” 2019, par. 210). This shows that what we call legal cognition, compared to legal reasoning, is about the articulation of the relation between substantial law (the rules) and the facts and can operate orthogonally of the reasoning about the validity of the evidence. The model that we present in Sect. 4 will be a model of substantial syllogistic reasoning – what we call legal cognition.

**Fig. 1:**
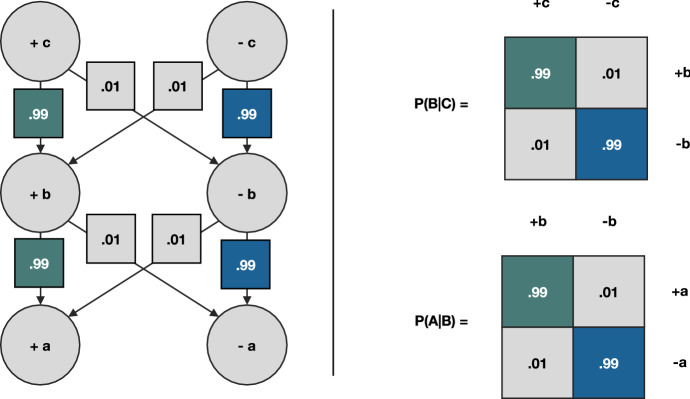
Tree like representation of the appellant’s argument in Pharmascience inc. **On the left-hand side**: The layers forming the tree-like structure represent the variables of the tree, which are A, B, and C. Each variable is set to be true ( +) or false (−). The tree unfolds in subsequent layers through edges, which represent the conditional probability of variable settings (e.g., – a conditioned on + b = 1%; + a conditioned on + b = 99%). The variables represent the elements of the proposition, and the squares on the edges represent the probabilities set by the logical implication. The probabilities come from the legal source itself. **On the right-hand side**: The matrices represent the conditional probabilities that make up the tree. “P” stands for “probability”, and the symbol “ |” stands for “conditioned upon”. For instance, the left matrix represents the probability (P) of the random variable A and its possible settings (+ a or − a) conditioned upon the random variable B and its possible settings (+ b or -b). This is written as P(A|B). Each column of the matrix must sum to 1, as each represents a conditional probability distribution

Depending on the domain of law, private or public, civil or criminal, the facts will be either event in a person’s life (e.g., an event causing an injury or the violation of contractual obligations) or an act of government or of the legislature (e.g., the adoption of a section of a statute). In Pharmascience inc. v. Option Consommateurs, the fact, or consequence was an act of the legislature (i.e., ss. 1002 of Quebec’s Code of Civil Procedure (C.c.p).), and the rules, or causes were other acts of the legislature (i.e., ss. 23 of the Canadian Charter of Rights). A complete legal syllogism such as the one of the appellants in Pharmascience inc. (unpacked in Table 1 above) will include subsidiary syllogisms, or even have premises that will themselves be made of syllogisms. For instance, the first proposition of the major of the appellant argument itself contained a syllogism, which was that class actions (A) require certification(Code of Civil Procedure 2016, ss. 575) (B) (if A then B), and that certification (B) involves a judicial decision (Code of Civil Procedure 2016, ss. 576) (C) (if B then C). Applying the logic of transitivity, the conclusion of that syllogism is that class actions involve judicial decision (A, then C). This syllogism captures the first proposition of the major of the appellant argument in Pharmascience inc. solely operates on rules as it moves from A to B, and from B to C based on Sects. 575 and 576 of Quebec’s Code of Civil Procedure (C.c.p.). No facts are involved in this syllogistic reasoning. This reflects the fact that the legal syllogism can include implication relations between facts and rules, and between rules of different orders (e.g., the relation between two sections of a statute, such as 575 and 576 C.c.p., or the relation between a legal precedent and a section of a statute that it interprets). In the syllogism above, each proposition of type A, B and C could be true or false (e.g., 575 and its underlying criteria may or may not apply). Thus, A could be + a (i.e., true) or -a (i.e., false), B could be + b (i.e., true) or -b (i.e., false), and C could be + c (i.e., true) or -c (i.e., false). All the possible permutations of the syllogism underwriting the first proposition of the appellant is presented in Table 2. Taken together, they trace a tree-like structure defining all the possible configurations of the argument formed by the legal syllogism (see Fig. 1.).

**Table 2:** Possible permutations of the first proposition

	Formal structure of the proposition	Elements of the proposition
Minor	If + a then + b	Class actions require certification
	If − a then − b	No class actions require no certification
	If + a then −b	Class actions require no certification
	If − a then + b	No class actions require certification
Major	If + b then + c	Certification involves a judicial decision
	If − b then − c	No certification involves no judicial decision
	If + b then − c	Certification involves no judicial decision
	If − b then + c	No certification involves judicial decision
Conclusion	If + a then + c	Class actions involve judicial decision
	If − a then − c	No class actions involve no judicial decision
	If + a then − c	Class actions involve no judicial decision
	If − a then + c	No class actions involve judicial decision

Now, it may be said that the syllogism is but one type of argument that can apply to computational models of legal argumentation. The notion of argumentation in social sciences (e.g., philosophy) refers to the process whereby parties will debate over which of the available positions is true relative to some standard regarding the meaning of truth. Argumentation in computational science and artificial intelligence has been defined as a process of logical reasoning whereby a conclusion is drawn from the most “acceptable” position out of a set of arguments and counterarguments (e.g., A is more acceptable than B). Acceptability can be weighted using preferences over arguments, which imposes a priority (Amgoud and Cayrol 2002). Preference or priority is a way to keep an argument in an acceptability class despite it being defeated by another argument over which we have negative preferences. For instance, if B defeats A, and C defeats B, but C is preferred to B, we might ignore the fact that A is defeated by B, and keep A as part of the acceptability class by letting go of B (Amgoud and Cayrol, 1998). Here we favor syllogistic reasoning as a structure of argumentation because the argumentative legal process does not involve “preferences” or “acceptability standards” as extrinsic leverage for drawing conclusions. Depending on the jurisdiction, preferences may be considered, though only over the interpretative process, under the form of standards for interpretations such as liberal vs. restrictive (e.g., living three constitutionalism in the jurisprudence of the Canadian supreme court (Miller 2011)). Legal argumentation is simply a process of “matching” the most probable evidenced facts with rules. Evidence, facts and rules are conditionally related to one another, using mappings that may be uncertain; hence the appeal in practice to legal interpretative methods (e.g., textual vs. teleological). In that sense, winning a legal argument just means discriminating between arguments based on the probability of those arguments “being the case”. In short, one does not need another legal argument (e.g., B) to decide whether an argument (e.g., A) should be rejected. In the law, when an argument wins, it is simply because the other falls.

As we will see in the next section, Bayesian approaches and an information-theoretic measure such as the entropy of conditional distributions, by providing intrinsic measures of how evidenced facts and rules “fit” together naturally lend themselves to the modelling and evaluating of legal arguments. That being said, this does not preclude other probabilistic approaches from being relevant to the modelling of legal arguments. In fact, many candidates are available that are looking at various credence score and degrees of beliefs over arguments (e.g., Hunter 2022; Haenni 2009). Such models ought to be considered in future work to evaluate which of the available approaches may be the most computationally efficient. Here, we opt for an implementation of the legal syllogism, first, as a tree like decision process, which we then model in Sect. 4 as a Bayesian network. As shown in Fig. 1, the tree is made of levels, which represent the elements of the proposition. These are Boolean random variables (i.e., A, B and C) whose setting can be “true” or “false” (e.g., A =  + a; − a). The edges that link the levels represent the probability of the variable below condition upon the variable above, or the probability of the variable below to be either true or false conditional upon the variable above being either true or false. When the tree is solely made of rules coming from sources such as statues, as is the case in Fig. 1, there is almost no uncertainty in the tree structure (i.e., 0.99 = 99%, 0.01 = 1%). This means that one can easily compute the most probable paths simply by looking at the tree-like structure. In Fig. 1, the conclusions of Table 2 are traced in blue and green.

Crucially, the paths in Fig. 1 should be read from the bottom to the top. For instance, the green path should be read as “if we observe + a, then + b will apply, and + c will be the case”, that is, following the appellant’s argument, if we observe a class action, this means that a judicial decision was made, and if a judicial decision was made, this means that the certification criteria were assessed. The tree-like representation inverses the direction of reasoning of the syllogism. The syllogism establishes a general rule, which is that A implies C because A implies B, and B implies C. When reasoning about the relation between A and C, one ought to invert, or retrace the path that led from A to C. Inverting the structure of the tree amounts to finding the most probable path and finding out which of the possible permutations of the argument will apply. Finding the correct permutation means solving the legal syllogism and corresponds to what judges and lawyers alike do when reasoning about the law.

**Fig. 2:**
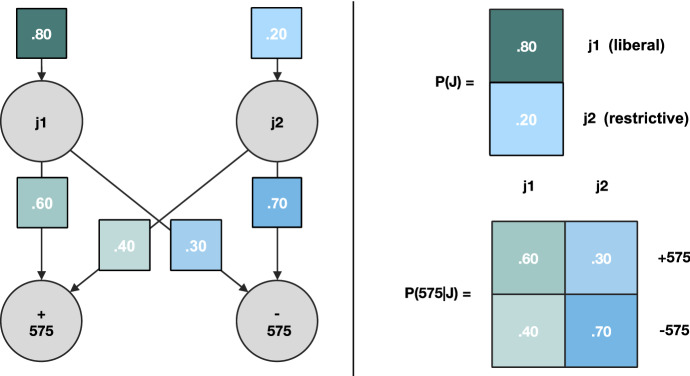
Probability of Sect. 575 C.c.p. and its jurisprudence. The random variable representing the jurisprudence “J” can take two settings, j1 and j2, which could stand for two different tendencies (e.g., liberal vs. restrictive). The random variable representing the Sect. 575 is “575” and is Boolean. It can take two settings, “true” ( +) or “false” (−). The edges represent the conditional probabilities in that form P(575|J), as well as the prior probability of the jurisprudence P(J). The prior probability corresponds to the probability of the jurisprudence, irrespective of the statutes conditioned upon it. For instance, in P(J), j2 being less probable than j1 could mean that there is a lesser amount of legal precedent using that interpretation. In the case of P(575|J), the differences in probabilities track the probability of an interpretation having been applied under 575 C.c.p.. The matrices encode all the probabilities, with the columns summing to 1

When the tree structure includes both rules and facts, this means finding the most probable path that leads from observable facts, or consequences at the bottom of the tree, to possible causes under the law, or rule moving upward in the tree. Legal syllogistic reasoning based on the tree-like structure of Fig. 1 is about finding “what rule will apply to the facts at hand, given that those facts were observed”. The first proposition of the major of the appellant argument in Pharmascience inc. was meant to establish the claim according to which “the institution of a class action requires a judicial decision”. This is obvious from the articulation of 575 and 576 C.c.p.. When operating solely based on clear statutory rules, there is no uncertainty in the conditional relations between the variables of the tree. We know that if Sect. 576 of the Civil Code of Quebec (C.c.Q.) is true, that is, if a certification ruling happened, we know, with almost 100% certainty, that the criteria for certification of article 575 C.c.Q. have been assessed, first. Oftentimes, syllogistic arguments will be used to demonstrate the applicability of a rule, per se. For instance, one could argue that the correct application of a criterion of 575 C.c.p. is conditioned on competing jurisprudential interpretations which may or may not allow 575 to apply given the facts at hand. That is, there may be uncertainty over whether a statutory rule such as 575 will apply, given the jurisprudence. Moreover, there could be uncertainty over which of the jurisprudential interpretations should apply, irrespective of the statute at hand (see Fig. 2).

Following Fig. 2, the jurisprudence “j1” might tend to interpret 575 C.c.p. liberally, which would suggest that the probability of 575 C.c.p. applying will be high (e.g., 0.6, or 60%), although j1 might still leave a 40% (0.4) probability of 575 C.c.p. not applying to the facts at hand. Conversely, a restrictive jurisprudence might tend to interpret 575 C.c.p. in such a way that its likelihood of applicability may be only of 30% (0.3), and of inapplicability of 70% (0.7). Which of the two interpretations j1 or j2 will apply can also be uncertain (e.g., 20% chance for j2, and 80% chance for j1). Taken together, the jurisprudential interpretations “J = j1; j2” of 575 C.c.p. will yield a scenario with much uncertainty.

In practice, judges, lawyers, and advanced students routinely deal with such uncertainty when they assess litigious situations. Indeed, the jurisprudence characteristic of common law legal systems is at its core an evolutionary process that yields competing interpretations of statutory law, which leads to uncertainty in the application of the law (Elliott 1985). Thus, any model of judicial reasoning, at least in common law systems, should model judicial reasoning not only as syllogistic reasoning, but also as syllogistic reasoning under probabilistic uncertainty. For this reason, in the next section, we propose a generic Bayesian model of syllogistic reasoning that shows how Bayesian models of legal reasoning can tackle probabilistic uncertainty stemming from competing jurisprudential interpretations of statutory law.

To help situate the reader, we should stress that the proposed model of legal syllogistic reasoning that we will discuss in the next section can be viewed as belonging to the more general class of probabilistic language models seeking to improve on first order propositional logic models. Although we start from a simple propositional logic interpretation of the legal syllogism, our end destination is a computational model that improves on first-order logic, where variables such as “A, B, and C” and their truth value will be modelled probabilistically (e.g., P(A), P(B), P(C)) within the context of a graphical model that puts these variables in relation to one another (e.g., P(A|B), P(B|C), etc.). This is nothing new, but here serves as a useful strategy for implementing legal syllogistic arguments into probabilistic Bayesian model of such arguments. In computer science, the motivation for moving to probabilistic language models is the limited applicability of first-order logic (and a fortiori propositional logic such as discussed above). This stems from the fact that first-order logic lacks semantics for plausible reasoning under uncertainty; first order logic being limited to variables that are either true, false, or indeterminate (Laskey 2008). Probabilistic logic models improving on first-order logic leverage properties such as independence assumptions of Bayesian graphical models, which render inference more efficient (e.g., Pearl 1988) and therefore accommodates some of the shortcomings of first order logic in computational theory (e.g., intractability of distributions needed to express truth values of a large number of assertions).

Accordingly, one natural route that this paper may have taken would have been that of seeking to better couch the model of the next section into the foundation of logic for language models improving on first-order logic with probabilistic inference (e.g., by grounding the proposed model into a First-order Bayesian logic (FOBL) model Laskey, (2008)). A second possible, though perhaps less intuitive route is that of framing the proposed model within the context of Bayesian approaches to cognition (Knill and Pouget 2004), therefore bringing this project somewhat out of the realm of computer science, and more into the domain of computational cognitive science. It is that second route that we explore in this paper.

## A Bayesian model of syllogistic reasoning

### Syllogistic reasoning as applied to jurisprudential interpretations, statutes and facts

This section offers a Bayesian interpretation of legal syllogisms under uncertainty. Our motivation for using a Bayesian method is that, as argued above, legal syllogistic reasoning is often done under uncertainty, and Bayesian methods are well suited to make inference under uncertainty. Within the context of legal proceedings, Bayesian methods are used to reflect the way in which one assesses the impact of evidence on the probability of a verdict (Fenton et al. 2013). Here, instead, we use Bayesian networks to model legal syllogisms under uncertainty. We propose a generic model that could apply to any legal syllogism. We will not base our model on a specific argument coming from the jurisprudence, as we did in Sect. 2 above to exemplify the formal structure of the legal syllogism articulating rules and facts. Rather, we will use a fictitious litigious scenario to show how rules such as jurisprudential interpretations influence the probability of the application of second order rules such as statutory law to facts.

Our scenario is as follows: Mr. Smith called Mr. David a liar. Mr. David wonders if he has a recourse against Mr. Smith in extracontractual liability under Sect. 1457 of the Civil Code of Quebec (C.c.Q.). 1457 C.c.Q. is the article that establishes the elements of torts, which are the fault, the injury, and causality. Mr. David has to show causality, fault, and injury. We assume that the causality and the fault do not need further demonstrations such that 1457 C.c.Q. will apply with respect to the facts in that regard. The debate is over whether the insult (i.e., being called a liar) can be a cause for injury. Our fictive scenario further assumes that there exists a fictive jurisprudence, which tends to follow two interpretations. One is a liberal interpretation under “Buller v. Alister”, which states that “an insult may be a cause for moral injury under 1457”, and the other is a restrictive interpretation under “Michael v. Adam”, which states that “an insult is not recognized as a cause for moral injury under 1457”. This reflects the fact that the application of statutes to facts sometimes requires an interpretation when the facts do not precisely fit the statute; an interpretation which is done using prior judicial decisions. Typically, one will find “liberal” and “restrictive” jurisprudential or statutory modes of interpretations; the liberal interpretation leaning on a wide application of the statute based on the purpose of the statute (i.e., teleological interpretation), and the restrictive interpretation leaning on a narrow application closer to the text (i.e., textual interpretation). The mode of interpretation can be codified or can be established by the jurisprudence. In Canada, for instance, the default mode of interpretation of statutory laws is the liberal mode of interpretation, which is codified in ss. 12 of Canada’s Interpretation Act of 1985.

We use Bayesian networks to model the causal structure of the possible syllogisms that one could derive from the above scenario. The legal syllogisms that we will model in this section rest on 3 random variables and their settings (see Table 3): (i) the fact variable “F” about lying and its settings, “ + f, -f”. The settings correspond to whether the fact will be qualifiable (+ f) or not (− f) as a cause for injury under the Sect. 1457 of the statute (S), which will be applicable (+ s) or inapplicable (-s), given the available jurisprudential interpretation of 1457 C.c.Q. (J). These are the liberal interpretation (j1) and the restrictive interpretation (j2). The remainder of this paper shows how one can model the syllogistic application of the law to the proposed fictive scenario.

**Table 3:** Random variables and settings

Random variables	Settings	Semantics
*J*: Jurisprudential interpretations	j1 = Michael	Liberal interpretation in the Michael case
	j2 = Buller	Restrictive interpretation in the Buller case
*S*: Statute’s applicability	+ s = + 1457	Applicability of 1457
	− s =− 1457	Inapplicability of 1457
*F*: Fact	+ f = insult is a cause − f = insult is not a cause	Mr. Smith’s insult as a cause for injury

### Bayesian networks

A Bayesian network is a computational strategy to quickly and efficiently represent and compute multiple priors and likelihood according to Bayes rule (see box 1). Bayesian networks essentially have the same structure as the tree-like representations described in Figs. 1 and 2. They are directed (i.e., flowing in one direction) acyclic (i.e., not involving feedback loops) graphs whose nodes correspond to random variables with edges that correspond to the parameters encoding the probability distributions linking the different nodes. When the structure of the model increases in complexity, the properties of directed acyclic graph become particularly relevant. Directed acyclic graphs allow one to identify which of the node is conditionally independent from the others (Pearl 1988). Given the simplicity of the proposed model, we will not need to exploit this property. We will simply use the Bayesian network to implement syllogistic legal arguments and compute the probability of each proposition (minor and major) and their entropy, or uncertainty (see box 1).

**Box 1:** Bayes rule and the meaning of uncertainty

So far, to capture the notion of uncertainty, we have used the intuition that a distribution with, say, two outcomes with probabilities of 0.99 (99%) and 0.01 (1%) was more certain than a distribution with two outcomes with probabilities of, say, 0.60 (60%) and 0.40 (40%). The intuitive or psychological understanding of uncertainty here is based on the fact that guessing the most probable observable outcomes under the first distribution would be easier than under the second distribution. The formal meaning of uncertainty that underwrites this intuition refers to Shannon entropy. Formally, Shannon entropy (H(X)) is the negative sum of probability outcomes, or settings of a random variable weighted by their log probability:	
$$H\left( X \right) = - \mathop \sum \limits_{i = 1}^{n} p_{i} \log 2\left( {p_{i} } \right)\qquad (1)$$	
Assuming a distribution P(A) with outcomes 0.99 and 0.01, and a distribution P(B) with outcomes 0.60 and 0.40, the uncertainties, or entropy (H) for these two distributions would be:	
$$H\left( A \right) = - \left[ {.99*\log 2\left( {.99} \right)) + \left( {.01*\log 2\left( {.01} \right)} \right)} \right] = .0808\qquad(2)$$	
$$H\left( B \right) = - \left[ {.60*\log 2\left( {.60} \right)) + \left( {.40*\log 2\left( {.40} \right)} \right)} \right] = .9710 \qquad (3)$$	
In eqs. 2 and 3 above, the entropy of P(A) (H(A)) is lower than that of P(B), which reflects the psychological understanding of uncertainty understood as the “unpredictability of an outcome”; the outcome of P(A) being easily predictable, and the outcome of P(B) being less easily predictable. In our Bayesian model of legal syllogistic reasoning, the entropy will be measured over the prior distribution of jurisprudential interpretations (P(J)) and the conditional distributions of facts on the statute (P(F|S)) and of the statute on the jurisprudential interpretations (P(S|J)). So far, we have also been silent on the formal meaning of probabilities such as P(J), P(F|S), P(S|J). In Figs. 1 and 2, based on intuition, we illustrated that these were “a priori” and “conditional” probabilities. The conditional probabilities were represented in a tree-like structure, with an arrow moving downward from what is on the right side of the condition symbol “ |” to what is on the left side of that symbol. More formally, a priori and conditional probabilities are known as the “prior” and the “likelihood” used in Bayes rule. Bayes rule is about establishing hypotheses about the relation between causes and consequences by inverting these causal relations to find the most probable cause of observed consequences. This is especially suited to situations where causes are unknown and where there is uncertainty as to which of those causes have generated the observed consequence at hand. Bayes rule states that the probability of a possible cause after having observed a possible consequence of that cause (P(cause |consequence), or “posterior distribution”) is equal to the joint probability of the cause and the consequence divided by the independent probability of the consequence. The join t probability is the prior probability of the cause times the probability of the consequence conditioned on that cause, or “likelihood (P(cause) X P(consequence | cause) = P(cause AND consequence). The independent probability of the consequence is the sum of the consequence conditioned on all possible causes weighted by the probability of the causes (P(consequence | cause_1) X P(cause_1) + P(consequence | cause_2) X P(cause_2):	
$${\text{P}}\left( {{\text{cause |cons}}.} \right) = { }\frac{{{\text{P}}\left( {{\text{cause AND cons}}.} \right)}}{{{\text{P}}({\text{cons}}.{ }|{\text{ cause}}\_1){\text{ X P}}\left( {{\text{cause}}\_1} \right){ } + {\text{ P}}({\text{cons}}.{ }|{\text{ cause}}\_2){\text{ X P}}\left( {{\text{cause}}\_2} \right)}} \qquad(4)$$	
Within the context of our syllogistic argument, P(J) is the independent, prior probability of the jurisprudential interpretations j1 and j2, whereas P(F|S) and P(S|J) are two likelihood probabilities + s and -s of the applicability of a statute (S) conditioned upon jurisprudential interpretations, and the probabilities + f and − f of the fact (F) conditioned on the statute (S) corresponding to 1457 C.c.Q.	

**Fig. 3:**
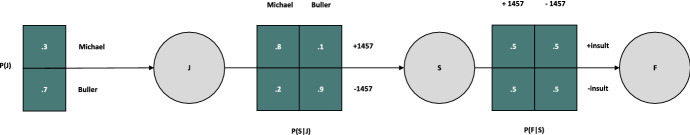
Bayesian network and model parameters based on the fictive litigious situation. This schematic represents a Bayesian network with 3 random variables F, S and J placed at the level of the nodes in the network (grey circles). The variables each have different settings, whose probabilities are represented by the edges (arrows) over which the network parameters (matrices) are placed. The model parameters encode the prior and conditional probabilities of settings in the network. From left to right, the first parameter P(J) is the prior probability over the jurisprudential interpretations (J) with settings “Michael” and “Buller”. The second parameter P(S|J) is the conditional probability of the settings (+ 1457; − 1457) of the statute (S) being applied conditioned upon the jurisprudential interpretations (P(S|J)). The third parameter is the conditional probability of the fact (F) with settings “ + insult;—insult” on the statute (S)

The Bayesian network of Fig. 3 below implements our fictive scenario. The model parameters are the matrices laid over the edges of the graph. The random variables J, S, and F are represented as nodes. We assume that the prior probability of the jurisprudence (P(J)) gives a 30% probability to the liberal interpretation under Michael v. Adam and the remaining 70% probability for the restrictive interpretation under Buller v. Alister. This would represent the fact that out of all the precedents having dealt with the qualification of insults under 1457 C.c.Q., 30% of the decisions have followed Michael v. Adam whereas 70% have followed Buller v. Alister; Buller v. Alister being the dominant jurisprudential interpretation. We further assume that the likelihood, or conditional probability (P(S|J)) of 1457 C.c.Q. being applied to cases of insults is of 80% under the Michael v. Adam interpretation and 10% under the Buller v. Alister interpretation. In turn, the probability of 1457 C.c.Q. not applying to cases of insults is of 20% and 90%, respectively. This reflects the fact that the applicability of statutes has various degrees of probabilities under the different jurisprudential interpretations.

Finally, we assume that the conditional probability of the fact being qualified or not under 1457 C.c.Q. (P(F|S)) is fully uncertain. There is an even chance (50%; 50%) that the fact at hand (i.e., lying) will be qualified as a cause of injury if 1457 C.c.Q. applies, or if 1457 C.c.Q. does not apply. Note that as it stands, the probability mappings in the node “F” provide no information, as the conditional probability is uniform, or flat (i.e., full uncertainty over how the facts should be qualified under Sect. 1457). Within the context of this model, this leads to a somewhat unusual situation where facts turn out to play no role in the syllogism. But that needs not be the case. The values of all the nodes in the proposed model have been selected to match the narrative that we use to illustrate how simple Bayesian networks may capture the structure of legal arguments and the syllogistic reasoning over them under various constrains imposed by the current state of the law (e.g., when one needs to appeal to statutory law and the jurisprudence to qualify facts that have never been qualified before). A distribution over the F node that would not be uniform would reflect the fact that alternative positions in the doctrine or the jurisprudence over whether “insults under 1457 can be a cause for injury” may exist. This may be the case, if this factual situation was treated by the Court across different cases without having been the object of a ratio decidendi, or if different positions on the question were discussed by the legal doctrine. For instance, the jurisprudence and the doctrine may be such that most of the time (e.g., 56% of the time) insult ought not to be treated as a potential cause for injury under the applications 1457 (i.e., + 1457). This would change the distribution of F, thereby impacting the rest of the syllogism. Here, we assume a F node that has no impact on the syllogism, thereby rendering the syllogism somewhat trivial.

Once the basic parameters of the Bayesian network are set, following Bayes rule, we can infer the missing parameters, which here would be the independent probability of the statute (P(S)) and the independent probability of the fact (P(F)). To do so, different methods are available. The simplest, though most computationally expensive method is known as the variable elimination method. Essentially, this method involves “joining” the prior and the likelihood probabilities, and eliminating the variables that are not needed to pursue the process by summing them out until the final distribution has been found, here P(F):

5 $$P\left( F \right) = \mathop \sum \limits_{S} P(F|S)\mathop \sum \limits_{J} P\left( J \right)P(S|J)$$

**Fig. 4:**
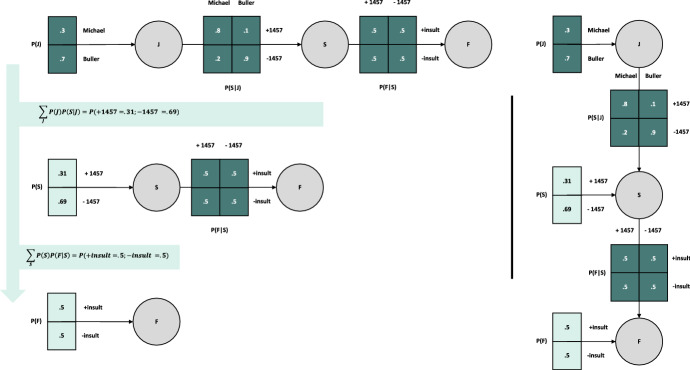
Step-by-step representation of variable elimination. The left panel represents the sum over the joint probability of the prior and the likelihood at each step, from top to bottom, to obtain the new prior probability of S and the evidence for F, both in light green. At the far right, we represent the Bayesian network with all its model parameters

By applying this method, one can find the missing parameters (light green, Fig. 4).

6 $$P{(}Micheal {|} + 1457) = \frac{{P{(} + 1457 {|} Micheal)P\left( {Micheal} \right)}}{{P\left( { + 1457} \right)}} = \frac{{\left( {.8*.3} \right)}}{.31} = .77$$

Once the missing model parameters have been inferred (light green on the right-hand side of Fig. 4), one can query the settings of interest under each variable and compute the posterior probability of one of the settings. For instance, if we are interested in the posterior probability of the Michael v. Adam’s interpretation conditioned upon the application of 1457 C.c.Q. (P(Michael |+ 1457)), we can compute its posterior probability following Bayes rule:

This can be done for all the posterior probabilities in the network (see Table 5 below). By listing all the posterior probabilities for each proposition of the syllogism (i.e., minors and major), one can identify which of the possible syllogistic arguments is the most probable. In our network, the most probable argument is “if Buller applies, 1457 does not apply (minor)”, and “if 1457 does not apply, insult is not a cause for injury” (major), therefore, “if Buller applies, insult is not a cause for injury” (7^th^ syllogism in Table 4). The minor of this argument has 91% probability, and the major of this argument has 69% probability.

**Table 5:** Table of syllogistic posterior probabilities

	Syllogism	Posterior probability	Graph
1	*Minor*		
	If Michael applies, 1457 applies	${\text{P (}}Micheal {|} + 1457) = \frac{{\left( {.3*.8} \right)}}{.31} = 77.$	
	*Major*		
	If 1457 applies, insult is a cause for injury	$P( + 1457| + insult) = \frac{{\left( {.31*.5} \right)}}{.5} = .31$	
	*Conclusion* If Michael applies, insult is a cause for injury		
2	*Minor*		
	If Michael applies, 1457 applies,	$P{(}Micheal {|} + 1457) = \frac{{\left( {.3*.8} \right)}}{.31} = .77$	
	*Major*		
	If 1457 applies, insult is not a cause for injury,	$P( + 1457| - insult) = \frac{{\left( {.31*.5} \right)}}{.5} = .31$	
	*Conclusion* If Michael applies, insult is not a cause for injury		
3	*Minor*		
	If Michael applies, 1457 does not apply,	$P(Micheal | - 1457) = \frac{{\left( {.3*.2} \right)}}{.69} = .09$	
	*Major*		
	If 1457 does not apply, insult is not a cause for injury,	$P( - 1457| - insult) = \frac{{\left( {.69*.5} \right)}}{.5} = .69$	
	*Conclusion* If Michael applies, insult is not a cause for injury		
4	*Minor*		
	If Michael applies, 1457 does not apply,	$P{(}Micheal {|} - 1457) = \frac{{\left( {.3*.2} \right)}}{.69} = .09$	
	*Major*		
	If 1457 does not apply, insult is a cause for injury,	$P( - 1457| + insult) = \frac{{\left( {.69*.5} \right)}}{.5} = .69$	
	*Conclusion* If Michael applies, insult is a cause for injury		
5	*Minor*		
	If Buller applies, 1457 applies,	$P{(}Buller {|} + 1457) = \frac{{\left( {.7*.1} \right)}}{.31} = .23$	
	*Major*		
	If 1457 applies, insult is a cause for injury,	$P( + 1457| + insult) = \frac{{\left( {.31*.5} \right)}}{.5} = .31$	
	*Conclusion* If Buller applies, insult is a cause for injury		
6	*Minor*		
	If Buller applies, 1457 does not apply,	$P{(}Buller {|} - 1457) = \frac{{\left( {.7*.9} \right)}}{.69} = .91$	
	*Major*		
	If 1457 does not apply, insult is a cause for injury,	$P( - 1457| + insult) = \frac{{\left( {.69*.5} \right)}}{.5} = .69$	
	*Conclusion* If Buller applies, insult is a cause for injury		
7	*Minor*		
	If Buller applies, 1457 does not apply,	$P(Buller | - 1457) = \frac{{\left( {.7*.9} \right)}}{.69} = .91$	
	*Major*		
	If 1457 does not apply, insult is not a cause for injury,	$P( - 1457| - insult) = \frac{{\left( {.69*.5} \right)}}{.5} = .69$	
	*Conclusion* If Buller applies, insult is not a cause for injury		
8	*Minor*		
	If Buller applies, 1457 applies,	$P(Buller | + 1457) = \frac{{\left( {.7*.1} \right)}}{.31} = .23$	
	*Major*		
	If 1457 applies, insult is not a cause for injury,	$P( + 1457| - insult) = \frac{{\left( {.31*.5} \right)}}{.5} = .31$	
	*Conclusion* If Buller applies, insult is not a cause for injury		

Once the posterior probabilities have been computed, one can further compute the entropy of the minor and the major (Table 6.). The most probable proposition should be sampled from the distribution for which we have the most certainty, that is, that has the least entropy. By computing the entropy of all the distributions, one can identify the distribution that contains the most probable argument. Finding the most probable propositions and articulating them under a legal argument is indeed what lawyers and judges do when they reason about the law, and as we have shown, can be replicated with Bayesian networks.

**Table 6:** Table of syllogistic uncertainty

	P(J |+ 1457)	P(J |− 1457)	P(S |+ f)	P(S |− f)
Distribution	P(Michael |+ 1457) = .77 P(Buller |+ 1457) = .23	P(Michael | -1457) = .09 P(Buller | -1457) = .91	P(+ 1457 |+ insult) = .31 P(-1457 |+ insult) = .69	P(+ 1457 |—insult) = .31 P(− 1457 |—insult) = .69
Entropy	.778	.436	.893	.893
Syllogisms	If Michael applies, 1457 applies	If Michael applies, 1457 does not apply	If 1457 applies, 1457 insult is a cause for injury	If Michael applies, insult is not a cause for injury
	If Buller applies, 1457 applies	If Buller applies, 1457 does not apply	If 1457 applies, insult is a cause for injury	If Buller applies, insult is not a cause for injury

## Conclusion

This paper proposed a Bayesian model of syllogistic reasoning that allows one to infer legal arguments under jurisprudential and statutory uncertainty. The proposed approach differed from standard Bayesian approaches to legal reasoning as its focus was on the modelling of the way practitioners and magistrates reason about the relation between rules and facts (i.e., a model of legal cognition) instead of on the modelling of how facts may be optimally proven using evidence and Bayesian inference. We first argued that legal reasoning followed a syllogistic form. We then unpacked the formal structure of the syllogism to make it amenable to modelling with Bayesian networks. Based on a fictive litigious situation from which multiple syllogisms could be derived to find whether “insult was a cause for injury”, we built a Bayesian network to assess the posterior probability of all those possible syllogisms and their uncertainty (Shannon entropy). Given the parameterization of our model, the most probable and the most certain syllogistic argument was indeed the one that one would have intuited; that is that “if Buller applies, 1457 does not apply (minor)”, and “if 1457 C.c.Q. does not apply, insult is not a cause for injury”, therefore, “if Buller applies, insult is not a cause for injury”. Of course, this is unsurprising, since we parameterized the model precisely to obtain such a result. The goal of this study was simply to propose a toy model of syllogistic legal reasoning based on Bayesian principles that would illustrate the way in which one could use Bayesian networks to accomplish standard reasoning tasks standard in legal practice.

Versions of the proposed model could become relevant to legal practice if designed to fit real litigious situations and if parameterized with data coming from the actual jurisprudence. Legal precedents for specific litigious situations could be used to parameterize the model parameters as we did, to find the most probable argument given the facts at hand. The goal would be to allow the model parameters to reflect the actual probabilities of legal precedents and statutes applying to a given factual scenario. One could further imagine the integration of such Bayesian model with legal research databases such as LexisNexis Advance Quicklaw which compiles the history of cases, citing cases, summaries, etc.; all of which are data sources that can be used to parametrize a Bayesian network to perform syllogistic reasoning under uncertainty.

Now, one may ask why a Bayesian model of syllogistic reasoning would be useful, given that legal practitioners spend years in higher education to become experts at performing legal reasoning. Experts might not need such a model to help them in their practice. However, this can be said of almost any domain of professional practice that seeks to integrate technologies to support the work of practitioners. The method developed in this paper is inspired by methods employed in another domain of professional practice – computational psychiatry (Huys, et al. 2016), where Bayesian models of cognition are used to develop tools to support the work of practitioners. In computational psychiatry, Bayesian models of cognition are developed to test the various effects of synthetic pharmacological and non-pharmacological interventions on simulated agents afflicted with different mental disorders. The hope is that these methods, for they provide a computational map of the causal relations between social and neurophysiological factors of mental disorders, may one day help psychiatrist with diagnostic nosology and prognostics by allowing for the testing, in silico, of hypotheses about the aetiology and about the effects of different modes of therapeutic interventions. Within the context of the law, more sophisticated versions of Bayesian models for legal cognition such as the one proposed in this paper may one day function as “maps of the law” and be used to assist the work of lawyers and judges by allowing for testing hypotheses about possible assemblages of facts and rules. By analogy, using those models, the kind of “diagnostic” one may pose on a legal system would rest on exploring “what would likely happen” to legal arguments and statutory interpretations if the judiciary branch or the legislative branch were to “intervene” on the parameters of the law (e.g., by changing the probability mappings in the statutory and the jurisprudential parameters through decisions and bills).

Alternatively, the proposed model could be used to improve technologies helping efforts towards access to justice by “smartening” currently available artificial intelligence solutions. In the region of Quebec, Canada, a good example of a technology improving access to justice is the JusticeBot of the “Tribunal Administratif du Logement” (housing tribunal). This bot is meant to simplify access to legal information in matter of residential leases. It operates based on queries that guide the user through an information path that helps the user in deciding over whether she should take legal actions given her factual situation. One can imagine an implementation of such information tools incorporating a Bayesian module guiding the user with probabilistic information about the chances of success of recourse, based on the current jurisprudence and other information parameterizing the module. Such a Bayesian module may allow one to qualify factual situations that go beyond low intensity disputes. Indeed, as mentioned in the introduction, and as illustrated in this paper, Bayesian approaches are well suited to perform inference under jurisprudential and statutory uncertainty. It is precisely this kind of uncertainty that legal professional must handle in high intensity disputes, when qualification standards become more open to interpretation (e.g., standards of reasonability) and when the jurisprudence presents a variety of possible interpretations.

All of those potential applications are of course speculations. The goal of the proposed model was simply to offer a proof of principle, or a first attempt at mapping concepts and processes that belong to legal analysis such as operated by practitioners onto a simple model. In that sense, the work done in this paper remains at the level of formal theory and proofs of principle.
